# cGMP signals in smooth muscle cells and cardiac myocytes

**DOI:** 10.1186/1471-2210-11-S1-P40

**Published:** 2011-08-01

**Authors:** Christian Krawutschke, Doris Koesling, Michael Russwurm

**Affiliations:** 1 Institut für Pharmakologie und Toxikologie, Medizinische Fakultät, Ruhr-Universität Bochum, 44780 Bochum, Germany

## Background

cGMP plays an important role in the cardiovascular system and is involved in smooth muscle relaxation and inhibition of platelet aggregation. cGMP concentrations inside cells are determined by an interplay of cGMP synthesis through natriuretic peptide or NO receptor guanylyl cyclases on the one hand and cGMP-degrading phosphodiesterases on the other hand.

Phosphodiesterase 5 has been suggested to play a role in cGMP hydrolysis in cardiac myocytes. Strong antihypertrophic effects of PDE5 inhibition by sildenafil have been observed in animal models and clinical trials have been started to approve sildenafil for the treatment of cardiac hypertrophy.

Nevertheless, cGMP increases induced by nitric oxide or natriuretic peptides in cardiac myocytes remain difficult to assess. Currently, cGMP levels are measured by radioimmunoassays (RIA) or enzyme-linked immunosorbent assays (ELISA) using cGMP-specific antibodies. Although femtomolar amounts of cGMP can be detected, relatively high agonist concentrations are required to obtain measurable cGMP increases.

## Results

Here, we measured cGMP elevations by fluorescence resonance energy transfer (FRET)-based cGMP indicators to compare cGMP signals in cardiac myocytes and smooth muscle cells.

